# **β**-Catenin Signalling in Glioblastoma Multiforme and Glioma-Initiating Cells

**DOI:** 10.1155/2012/192362

**Published:** 2012-02-12

**Authors:** Mireia Nager, Deepshikha Bhardwaj, Carles Cantí, Loreta Medina, Pere Nogués, Judit Herreros

**Affiliations:** ^1^Departments of Basic Medical Sciences and Experimental Medicine, IRBLleida University of Lleida, 25198 Lleida, Spain; ^2^Neurosurgery Unit, University Hospital Arnau de Vilanova, 25198 Lleida, Spain

## Abstract

Glioblastoma multiforme (GBM) is a commonly occurring brain tumor with a poor prognosis. GBM can develop both “de novo” or evolve from a previous astrocytoma and is characterized by high proliferation and infiltration into the surrounding tissue. Following treatment (surgery, radiotherapy, and chemotherapy), tumors often reappear. Glioma-initiating cells (GICs) have been identified in GBM and are thought to be responsible for tumors initiation, their continued growth, and recurrence. **β**-catenin, a component of the cell-cell adhesion complex and of the canonical Wnt pathway, regulates proliferation, adhesion, and migration in different cell types. **β**-catenin and components of the Wnt canonical pathway are commonly overexpressed in GBM. Here, we review previous work on the role of Wnt/**β**-catenin signalling in glioma initiation, proliferation, and invasion. Understanding the molecular mechanisms regulating GIC biology and glioma progression may help in identifying novel therapeutic targets for GBM treatment.

## 1. Introduction

Gliomas are the most common primary malignancies in the central nervous system, comprising a heterogeneous group of tumors that display some histological similarities to glia (mainly, astrocytes in astrocytomas and oligodendrocytes in oligodendrogliomas). Astrocytomas account for the majority of gliomas, which can be classified into four different grades according to the World Health Organization (WHO) classification system [1, 2]: grade I and grade II astrocytomas are slow-growing less aggressive tumors, whereas grade III and IV gliomas are malignant tumors characterized by high proliferation rate (grade III) and the presence of necrotic tissue and/or angiogenic activity (grade IV). The most malignant form, glioblastoma multiforme (GBM, grade IV), is one of the most aggressive and lethal forms of cancer with an average survival time of 15 months after diagnosis [3, 4]. Standard treatment consists of surgical removal of the tumor, followed by chemotherapy and radiotherapy. Temozolomide, an oral alkylating agent, is the most commonly used chemotherapy treatment [5]. Importantly, a high infiltration capacity of individual cells over long distances, already present in grade II gliomas, hinders complete tumor resection and most likely contributes to recurrence [6]. 

GBMs can present as primary or secondary. Primary or “de novo” GBMs, representing the majority of GBM cases, arise without any prior evidence of a lower-grade precursor lesion and more commonly affect older patients (mean age of 62 years). Secondary GBMs progress from a lower-grade glioma and typically develop in younger patients (median age of 45 years). Gliomas exhibit a vast array of genetic changes that contribute to the malignant phenotype [6–10]. These include loss of function mutations in the p53 tumor suppressor and hyperactivation of receptor tyrosine kinase (RTK) signalling, such as epidermal growth factor receptor (EGFR), platelet-derived growth factor receptor (PDGFR), and the receptor for hepatocyte growth factor c-Met. The signalling cascades downstream of activated RTK often result in activation of Ras and AKT. Mutations in tumor suppressors such as phosphatase and tensin homolog (PTEN) and neurofibromatosis 1 (NF1) that normally control these pathways further contribute to oncogenesis [2, 3, 6–10]. Based on its gene expression profile, GBM can be further classified into proneural, neural, mesenchymal, and classical types [8, 9]. This classification should allow a molecular stratification of GBM cases with important therapeutic implications. 

Here we focus our attention on Wnt/*β*-catenin signalling, a pathway primarily involved in embryogenesis and displaying important functions in adulthood when aberrant Wnt signalling has been linked to disease and cancer [11–14]. Understanding how *β*-catenin signalling regulates gliomagenesis and tumor progression may lead to novel therapeutic interventions in GBM. Firstly, therefore, we discuss the role of glioma-initiating cells (GICs), a type of “cancer stem cells,” in glioma development and evidence suggesting that Wnt/*β*-catenin signalling regulates GICs biology. We then review progress in the understanding of the involvement of Wnt/*β*-catenin signalling in the proliferation and invasion of glioma tumor cells.

## 2. Glioma-Initiating Cells (GICs) and Signalling Pathways in GICs

The existence of brain tumor stem cells was proposed about a decade ago, following advances in the stem cell field and the discovery that neurogenesis persists in the adult brain [15–18]. Glioma-initiating cells (GICs) share the features of neural stem cells that have been identified in GBM, including the expression of CD133 (prominin), the ability to form neurospheres, and the reproduction of tumors [19, 20]. However, the role of CD133+ and CD133− GIC subpopulations in tumor initiation is not clear. CD133− cells from the C6 glioma cell line showed clonogenic, self-renewal, and tumorigenic capacities [21]. Nonetheless, CD133− GICs isolated from primary GBM were as capable of producing tumors as CD133+ cells [22]. Individual GBM may contain CD133+ and CD133− GICs that represent different stages of differentiation [23]. Furthermore, multipotent CD133+ GICs contain a CD144+ (vascular endothelial cadherin positive) subpopulation that can give rise to tumor endothelial cells [24].

Signalling by several morphogens and cytokines (including leukemia inhibitory factor (LIF), fibroblast growth factor (FGF) and members of the Wnt, transforming growth factor-*β* (TGF-*β*)/bone morphogenetic proteins (BMP) families) maintains the self-renewal capacity of embryonic stem cells and supports cancer stem cell growth [25]. Thus, TGF-*β* signalling through the induction of LIF and the JAK-STAT pathway promotes the self-renewal of patient-derived GICs [26]. Seoane and coworkers recently identified a population of CD44 high/Id1 high GICs in GBM that locates in the perivascular niche. Depletion of this cell population by TGF-*β* inhibitors prevents tumor initiation and recurrence. This work identifies CD44 and Id1 levels as prognosis markers in GBM and shows that TGF-*β* signalling is key to maintain this GIC population [27]. Aberrant activation of sonic hedgehog signaling (another morphogen involved in embryogenesis and brain development) in committed cerebellar granule neuron precursors is responsible for aggressive medulloblastoma, a pediatric cerebellar tumor [28–30]. Hedgehogs signal through Gli transcription factors, with Gli1 and Gli2 acting as activators and Gli3 as a repressor factor. Consistent with the isolation of Gli1 from glioma cells, activation of the Hedgehog-Gli1 pathway is reported in GBM, which is required for the clonogenicity and formation of secondary neurospheres of CD133+ GICs [31].

**Figure 1:**
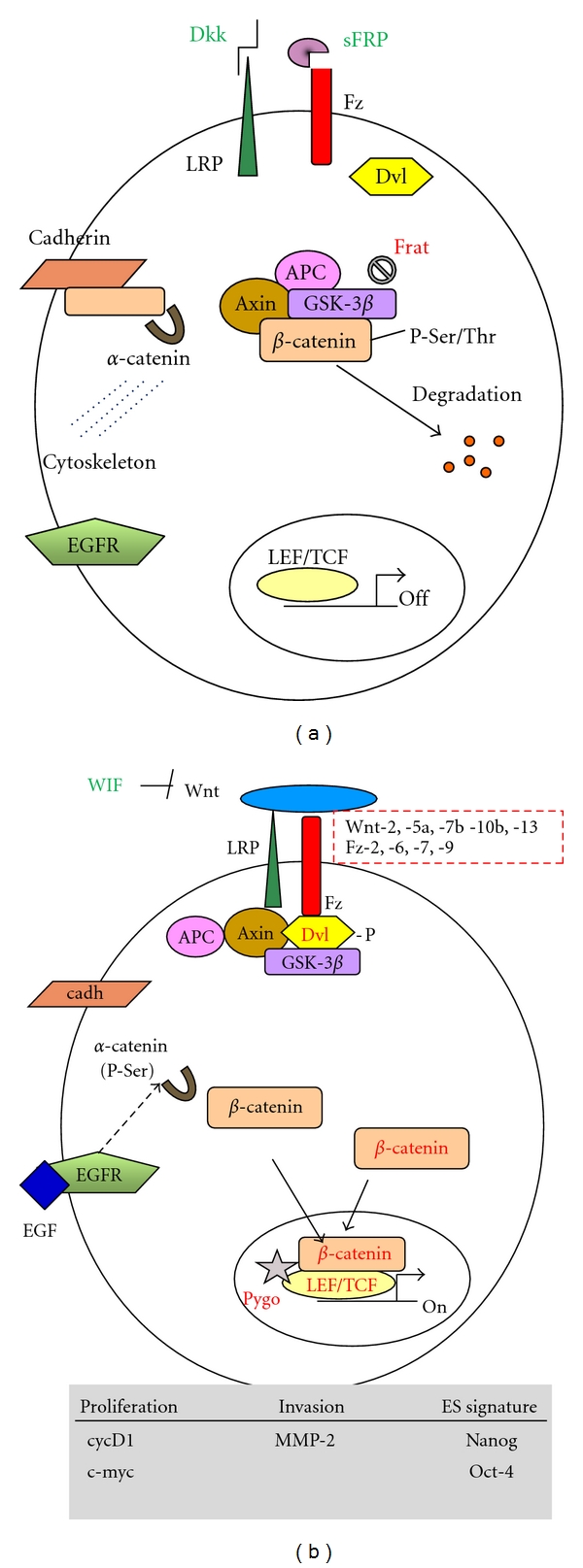
Wnt/*β*-catenin signalling and *β*-catenin role in adhesion in glioma cells. A) In the absence of Wnt ligands or in the presence of Wnt antagonists Dkk and sFRP that bind to the respective Wnt receptors Fz and LRP5/6, *β*-catenin is in a complex together with Axin, APC, and Gsk-3*β*. Here, *β*-catenin is phosphorylated by Gsk-3*β* in key Ser and Thr residues and is thus targeted for proteasomal degradation. Frat prevents the phosphorylation of *β*-catenin. Transcription by Lef/Tcf is off due to the binding of repressors. In the absence of growth factor signaling, a pool of *β*-catenin is engaged in the cadherin/*β*-catenin/*α*-catenin complex that is linked to the cytoskeleton. B) Following Wnt binding, the Fz-LRP5/6 complex is formed upon Dvl phosphorylation that recruits Gsk-3*β*, Axin, and APC to the membrane. This results in free *β*-catenin that accumulates in the cytosol and translocates to the nucleus, where it binds to Tcf and recruits transcriptional activators (including Pygo). Lef/Tcf transcriptional activation results in the regulation of Wnt target genes. The box shows Wnt target genes implicated in proliferation and invasion of glioma cells or conferring ES cell signature to GICs that might be related to aggressive growth and recurrence [37–39] EGF signalling through EGFR, ERK1/2, and CK2 results in the phosphorylation of *α*-catenin and promotes *β*-catenin transactivation [40]. Whether the Wnt-induced and growth factor-induced *β*-catenin nuclear pools collaborate in glioma cells remains to be studied. Text in red indicates Wnt pathway components that are overexpressed and green indicates Wnt antagonists repressed in high-grade astrocytomas and GBM. Wnt factors and Fz that have been reported to be upregulated in high-grade astrocytomas and GBM are shown (see references in the text).

Wnt factors are a family of secreted glycoproteins (19 members exist in humans) that regulate embryonic patterning and play different roles throughout development of the nervous system [32, 33]. Wnts signal through at least three different pathways [11, 33], the best known being the Wnt/*β*-catenin canonical pathway (Figure 1). In the absence of Wnt, the Ser/Thr kinase glycogen-synthase kinase (Gsk-3*β*) in the so-called destruction complex (comprising of Gsk-3*β*, adenomatosis polyposis coli (APC), Axin and *β*-catenin) phosphorylates *β*-catenin, which is then targeted for proteasomal degradation. Upon Wnt binding to Frizzled (Fz) (of which there are 11 family members in humans) and low-density-lipoprotein-related protein (LRP)5/6 receptors, the scaffolding protein Dishevelled (Dvl) and LRP5/6 become phosphorylated by Gsk-3 and Casein-Kinase I*γ*. Consequently, the destruction complex components are recruited instead to the receptor complex, leading to *β*-catenin stabilization [34]. The protooncogene Frat/GBP further prevents the phosphorylation and degradation of *β*-catenin because it competes with Axin to bind Gsk-3 and removes it from the destruction complex. Stabilized *β*-catenin translocates to the nucleus, where it binds to lymphoid enhancer factor-1 Lef-1/T-cell factor (Tcf) transcription factors and regulates expression of Wnt target genes. In the absence of nuclear *β*-catenin, Tcf/Lef factors suppress the expression of target genes through their binding to members of the Groucho/transducin-like enhancer of split (TLE) family of transcriptional corepressors. *β*-catenin does not have a DNA binding domain but it has a potent transcription activation domain. Conversely, Lef/Tcf transcription factors do not have a strong transcription activation domain, but they do have a good DNA binding/bending domain [35]. Thus, when *β*-catenin binds to a Lef/Tcf protein, a potent transcription regulatory complex is formed. Nuclear translocation of *β*-catenin converts Tcf proteins into potent transcriptional activators by displacing Groucho/TLE and recruiting an array of coactivator proteins, including CBP, TBP, BRG1, BCL9/PYGO, Legless, Mediator, and Hyrax [36] (Figure 1). 

Canonical Wnt/*β*-catenin signalling is crucially involved in embryonic development and controls stem cell biology, thus inducing self-renewal properties in embryonic stem (ES) cells and regulating adult stem cells [11, 14, 33, 41–45]. Nanog and Oct-4, two of the four transcription factors required to generate the pluripotency and self-renewal of ES cells, are Tcf3 targets [46, 47]. Interestingly, the ES signature characterized by the expression of Nanog, Oct-4, Sox-2, and c-Myc also associates with aggressive tumors, including poorly differentiated GBM [37]. As aberrant activation of the Wnt/*β*-catenin signalling pathway is a hallmark of many tumors [12, 14], these findings suggest that Wnt-regulated genes may contribute to the stem cell-like phenotypes displayed by brain tumors. Furthermore, the novel protooncogene *PLAG2* is amplified in GBM and promotes GICs proliferation and gliomagenesis. PLAG2 increases the expression of Wnt-6, Fz-9, and Fz-2, inhibits differentiation, and increases proliferation of neural progenitors [48]. It is worth noting that *PLAG2* amplification correlates with increased *β*-catenin levels in GBM samples. These results indicate that PLAG2 imparts stem-cell properties to glioma cells by regulating Wnt signalling. Another gene regulating Wnt signalling in glioma is *PEG3 *(paternally expressed gene 3), an imprinted gene with a tumor suppressor activity. Hypermethylation of *PEG3* promoter in glioma decreases *PEG3* mRNA expression and correlates with high-grade gliomas [49]. In turn, low PEG3 expression increases *β*-catenin that promotes the proliferation of CD133+ GICs [49]. Finally, the interaction between the transcription factor Forkhead box M1 and *β*-catenin that promotes *β*-catenin nuclear localization in tumor cells and maintains GIC self-renewal has been recently described [50]. Novel therapeutic interventions for GBM could inhibit Wnt/*β*-catenin signalling in GICs to decrease GIC proliferation and stop glioma growth, while increasing GICs differentiation.

## 3. Targeting GIC Chemoresistance and Radioresistance Mechanisms as an Approach to Treat GBM Recurrence

Following surgery, chemotherapy, and radiotherapy GBM recurrence is common. Therefore, a particularly relevant feature of the cancer stem cells is their ability to export drugs and develop resistance mechanisms to cytotoxics and irradiation [17]. Current knowledge suggests that resistance to temozolomide is promoted by enhanced O-6-methylguanine-DNA-methyltransferase- (MGMT-) mediated DNA repair of mismatches [51]. Thus, *MGMT *promoter methylation status improves the benefits of chemotherapy. According to Liu eta l., CD133 expression in tumor tissue is higher in recurrent GBM than in newly diagnosed tumors and CD133+ GICs are chemoresistant to temozolomide [52]. However, these findings are inconsistent with a report indicating that CD133+ GICs are depleted after temozolomide treatment [53]. Consequently, at present it is not clear which glioma cells are responsible for the resistance to temozolomide.

Another mechanism of drug resistance is the expression of the ATP-binding cassette (ABC) transporters by cancer stem cells. BCRP1 and MDR1 ABC transporters allow the exclusion of Hoechst 33342 dye, a feature that defines the pluripotential side population (SP) originally reported in haematopoietic stem cells and now used to identify cancer stem cells. Expression of these ABC transporters accounts for the chemoresistance of some cancers and high drug efflux capacity. CD133+ GICs express higher levels of BCRP1 compared to CD133− cells [52]. Consistent with this, a cancer stem-cells cell line (WJ_2_) derived from GBM showed increased expression of BCRP1, CD133 and the neural precursor marker Nestin and at the same time maintained Wnt-1 expression [54]. Interestingly, overexpression of MDR1 downstream of Wnt-1/Fz-1 signalling mediates chemoresistance in neuroblastoma cells [55]. This suggests that a similar mechanism could be operating in the chemoresistance mechanisms of gliomas.

As regards radioresistance, the CD133+ stem cell fraction is enriched after glioma radiation [56]. Furthermore, the CD133+ subpopulation is able to repair radiation-induced DNA damage more efficiently than CD133− tumor cells [57]. These results indicate that the CD133+ tumor cell population confers radioresistance to GBM and most likely accounts for glioma recurrence. Wnt-1 ectopic expression triggers DNA damage response in epithelial mammary cells [58], while activation of Wnt/*β*-catenin signalling confers radioresistance to mammary progenitors cells through survivin upregulation [59]. In addition, Gsk-3*β* inhibition enhances DNA repair of double-strand breaks following radiation of hippocampal neurons [60]. Taken together, these findings suggest that Wnt signalling may be involved in the chemo- and radioresistance mechanisms developed by GICs. Expanding our understanding of the molecular mechanisms supporting GICs resistance to conventional glioma treatment will allow the design of novel therapeutic tools to decrease tumor recurrence and improve patient survival.

## 4. Wnt/*β*-Catenin Signalling in the Proliferation of Glioma Cells

GICs represent a small percentage of the brain tumor mass, which is thought to contain a heterogeneous mixture of tumor cells with limited proliferation capacity. Molecular analysis on whole tumor samples is expected to mainly represent non-GIC cells. Wnt/*β*-catenin signalling plays a role in the proliferation of glioma tumor cells and tumor progression. *β*-catenin has been proposed as a prognostic marker in GBM, as both mRNA and protein levels increase in high-grade astrocytomas and GBM, thus correlating with malignancy [38, 61, 62]. In addition, the expression of other positive regulators of the Wnt pathway (including Dvl-3, FRAT-1, Pygo-2, Tcf4, and Lef-1) [38, 63, 64] and of Wnt target genes (namely the regulators of cell proliferation *Cyclin D1 *and* c-myc) *[38, 62] also increases in high-grade astrocytomas and GBM (see Figure 1). Using immunohistological techniques, a nuclear fraction of *β*-catenin was observed that associates with high-grade astrocytoma and GBM [62]. This result suggests increased cytoplasmic stabilization of *β*-catenin that escapes proteasomal degradation, in addition to the elevated *β*-catenin mRNA levels reported in GBM. Silencing *β*-catenin, Wnt-2, and Pygo-2 expression demonstrated the involvement of Wnt/*β*-catenin signalling in the proliferation of U251 glioma cell line [63, 65]. Together, these findings point to the activation of nuclear *β*-catenin signalling as a mediator of Wnt-induced proliferation of glioma cells. Moreover, expression of noncanonical Wnt-5a is also upregulated in high grade gliomas, in which Wnt-5a stimulates cell proliferation [66]. Wnt-5a signalling can inhibit canonical Wnt signalling during development [67, 68]. How Wnt canonical and noncanonical pathways interact in glioma cells remains to be studied.

In contrast to other cancers, no mutations have been found in *β*-catenin exon 3, a hot spot affecting the GSK-3 phosphorylation sites and *β*-catenin degradation that renders *β*-catenin active in glioma samples and cell lines [69, 70]. Truncation of APC, a mechanism causing polyposis and predisposing for Wnt/*β*-catenin-driven colorectal carcinoma, has not been associated with gliomagenesis (with the exception of Turcot syndrome patients) [71]. These observations suggest the deregulation of the pathway by unbalanced ligand/antagonist expression during tumor initiation and progression. Indeed, in addition to regulation of the expression of Wnt family members, Wnt antagonists often appear repressed in GBM (Figure 1). Expression of the Wnt antagonist and tumor suppressor Wnt inhibitory factor (WIF) decreases with malignancy in astrocytomas, which has been linked to aberrant promoter hypermethylation [72]. Also, hypermethylation of the *secreted-Frizzled-related protein* (*sFRP*) promoters is a significant event in primary “de novo” GBM, whereas hypermethylation of the promoter of the LRP antagonist *Dickkopf *(*Dkk*) associates with secondary GBM [70]. Similar epigenetic modifications are common to other Wnt-driven cancers [73, 74]. In addition, a novel mechanism for *β*-catenin nuclear localization and transcriptional activation (both constitutive and Wnt-induced) that controls Wnt target gene expression and glioma tumorigenesis has been described, which involves the interaction of *β*-catenin with FoxM1 [50].

## 5. *β*-Catenin and Wnt Signalling in Glioma Invasion

As a component of the cell adhesion complex, *β*-catenin binds to cadherin, thus regulating cell-cell adhesion. Altering the binding of *β*-catenin to cadherin or to *α*-catenin downregulates cell adhesion, while promoting cell migration and epithelial-mesenchymal transition [75]. However, *β*-catenin nuclear signalling is not only achieved by Wnt factors in tumor development [76]. Growth factor signalling can induce the phosphorylation of specific tyrosine residues of *β*-catenin, resulting in increased migration [75, 77–79]. EGFR expression is upregulated in primary GBM correlating with malignancy [15]. EGF/EGFR signalling through extracellular signal-regulated kinases 1/2 (ERK1/2) and casein kinase-2 (CK2) in glioma cells results in the phosphorylation of *α*-catenin at serine 641, which correlates with glioma malignancy [40]. Interestingly, *α*-catenin phosphorylation promotes *β*-catenin transactivation and glioma cell invasion [40]. These results highlight the involvement of *β*-catenin signalling not only as a mediator of Wnt but also downstream of growth factor signalling in glioma invasion. On the other hand, enhanced expression of the Fz antagonist sFRP2 reduced glioma invasion by decreasing *β*-catenin tyrosine phosphorylation and downregulating matrix metalloprotease-2 (MMP-2) [39]. However, sFRP2 did not affect *β*-catenin levels, its cytoplasmic/nuclear distribution, or its serine phosphorylation status [39]. How sFRP2 signalling modulates *β*-catenin tyrosine phosphorylation requires further investigation.

Noncanonical Wnt-5a, which signals through *β*-catenin independent pathways (including the planar cell polarity and the calcium pathways [33]), enhances the migration of glioma cells by regulating the expression of MMP-2 [80]. Moreover, silencing the expression of Wnt-2, Wnt-5a, and Fz-2 in the U251 glioma cell line decreases invasion [65, 80]. These findings are consistent with Wnt-5a function in invasion and metastasis in other cancers [11, 81, 82]. Together, these results point to metalloprotease regulation as important downstream targets of *β*-catenin and Wnt signalling pathways in glioma invasion [39, 80, 83].

We are accumulating knowledge on the signalling pathways responsible for the maintenance of GICs, sustaining the proliferation of bulk tumor cells and dictating the invasive properties of glioma cells. Current and future research should offer novel opportunities for anticancer drug discovery. Undoubtedly, the cancer stem cell hypothesis has provided a promising framework for investigations into an incurable disease. Future combined therapies including cytotoxics, tumor-targeted drugs, and agents that target GICs should be expected to reduce glioma growth and recurrence, raising hopes for glioma patients.
